# Perioperative and Postoperative Outcomes of Proximal Hip Fracture: A Comparison of Orthopedic and Geriatric Care Models

**DOI:** 10.7759/cureus.65899

**Published:** 2024-07-31

**Authors:** Omri Lubovsky, Philip J Rosinsky, Rimon Artoul, Dana Avraham, Maria Oulianski

**Affiliations:** 1 Orthopedics, Barzilai Medical Center, Ashkelon, ISR; 2 Geriatrics, Barzilai Medical Center, Ashkelon, ISR; 3 Orthopedics, Kaplan Medical Center, Rehovot, ISR

**Keywords:** orthogeriatric, geriatric trauma, orthogeriatric care models, proximal femur fracture, geriatric hip fracture

## Abstract

Introduction

Orthogeriatric patients with femur fractures, despite their multiple comorbidities and associated healthcare costs, have a promising new approach. This approach suggests that most patients should be hospitalized in the geriatric department, with daily orthopedic follow-up. The potential for lower mortality rates through orthogeriatric co-management and dual care is a reason for hope in our field.

Methods

This study is retrospective and involved 285 patients with proximal hip fractures. Two treatment models were compared: hospitalization in orthopedic and geriatric departments with different treatment protocols. The study analyzed demographic data and postoperative outcomes. It also included an analysis of 26 patients who received conservative treatment.

Results

Our study revealed significant differences between patients hospitalized in the orthopedic and geriatric departments. Geriatric department patients, who were significantly older and had higher comorbidities, experienced extended hospitalization and higher mortality rates during hospitalization, at 30 days, and at one-year follow-up (p<0.05). Notably, a significantly higher proportion of geriatric patients were discharged to home rehabilitation at the end of hospitalization compared to orthopedic patients (17.5% vs. 7.4%; p<0.01). Among non-operated patients, the mortality rate was 57.7% compared to 16.5% in patients who underwent surgery during the one-year follow-up.

Discussion

Our study suggests that elderly patients with hip fractures may benefit from management in the geriatric department. Despite experiencing significantly longer hospital stays, these patients have a higher likelihood of being discharged home compared to those managed in the orthopedic department. These findings have important implications for the care of orthogeriatric patients and may help guide future treatment strategies.

## Introduction

Hip fractures are common orthopedic trauma in elderly patients. Most of the patients fall in the category of ortho-geriatric patients, with ages above 65; the mean age is 75 to 79 years [1,2]. The socioeconomic costs of treating hip fractures in older people represent 0.1% of global healthcare costs worldwide [2]. These patients often have multiple comorbidities and disabilities and are associated with increased morbidity and mortality, increasing healthcare costs and burden on the system [3]. Health systems are in a continuous struggle. Urgent new approaches are needed for accommodation and the ability to provide adequate quality of care service. Proximal hip fractures are different in men and women with aging; two-thirds of hip fractures occur in women, which may be attributed to higher rates of osteoporosis in females [4,5]. However, the mortality risk is higher in men [6,7]. Annual mortality estimation suggests that 20-30% of deaths are directly related to hip fractures [8]. The one-year mortality rates in conservative treatment are around 60% and 16-33% in patients who underwent surgery [9-11]; after their release from the hospital, many remain in nursing homes or require long-term rehabilitation [12,13].

Perioperative management of elderly hip fracture patients is complex and different from that of young orthopedic trauma patients. Geriatric medicine has a holistic care approach for these complex patients. The involvement of geriatric specialists may significantly enhance the care for these patients during and after hospitalization. The promising approach of orthogeriatric co-management, a collaboration between orthopedic surgeons and geriatric teams, has recently gained traction and has been validated by a meta-analysis [14]. This dual care for these patients has the potential to greatly improve the overall outcomes [14,15], offering a hopeful prospect for the future of patient care. However, the best model of care currently remains uncertain [16].

Elderly patients with hip fractures are admitted to various departments of the hospital. Upon arrival at the emergency department, most patients are admitted to the hospital's orthopedic, geriatric, and other wards [1]. The primary purpose of this study, which holds significant implications for the future of patient care, was to compare the outcome of elderly hip fracture patients admitted to orthopedic and geriatric departments by quantifying indicators such as mortality, length of hospitalization, time to surgery, and other factors. This comparison will enable us to establish new and improved standards of care in our hospital, thereby enhancing the quality of service we provide.

## Materials and methods

**Figure 1:**
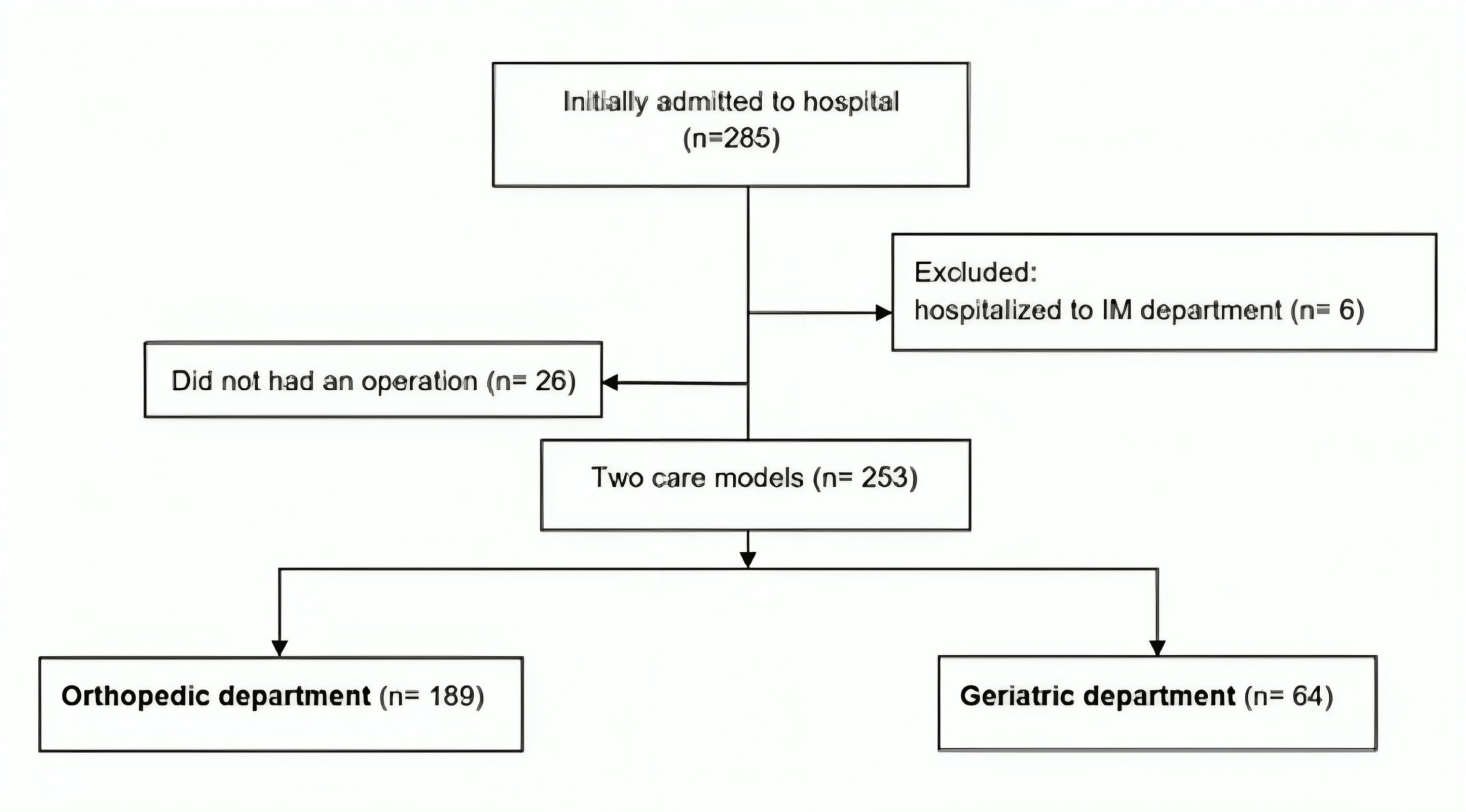
Flowchart for the selection and inclusion of the study IM - internal medicine

The present study is a retrospective cohort study. Data were extracted from medical records of 285 consecutive proximal hip fracture patients who presented to the emergency department (ED) from a university-affiliated hospital in Israel between October 2019 and December 2020. Patients were selected for inclusion based on the diagnosis of hip fracture using the International Classification of Diseases, 9th edition (ICD-9) diagnosis of hip fracture (820.xx). Some patients, usually living alone, are admitted with a delay, even a few days after the injury. Patients were included in the study only if the injury was during the first 24 hours before the admission. The electronic patient files were searched, and the following demographic data were obtained: age, gender, place of residence, marital status, department of hospitalization, length of hospitalization, mortality, discharge destination, and others. Based on radiographic images upon presentation, the fracture type was classified as a transcervical, base of neck, or pertrochanteric fracture. Patients were included if they presented with the aforementioned fractures and were over 67 years of age. We split the study group into two main models based on the hospitalization department (orthopedics and geriatrics). The patients who were not operated on had also been analyzed. Details of the selection process are shown in Figure 1.

Model of treatment

We have created a treatment algorithm in which elderly patients with hip fractures were admitted from the ED to the geriatric or orthopedic department based on available hospitalization beds. Unstable elderly hip fracture patients were admitted to the internal medicine department.

In the orthopedic department group, patients received continuous care in the orthopedic department until they were ready for transfer to a rehabilitation or other department. The geriatric consultative service was available on request, ensuring the continuity of care throughout the patient's journey.

In the geriatric department group, patients were treated in the geriatric department. There was orthopedic daily consultation service and examination of the patient until discharge.

The rehabilitation protocol in both departments was initiated on the first postoperative day, demonstrating our proactive approach to patient care. It continues with one physiotherapist workup per day with the same group of physiotherapists and other supportive treatment care groups.

Data analysis

Categorical variables were reported as frequencies and percentages and compared using Pearson's Chi-squared test. Continuous variables were reported as a median and interquartile range, or mean and standard deviation, and compared using the Mann-Whitney U test or analysis of variance (ANOVA), as appropriate. A p-value <0.05 was considered statistically significant. Data analysis was performed using SPSS for Windows (version 25.0; IBM Inc., Armonk, New York). Diagrams of curves were drawn with the use of Excel (Version 2101; Microsoft, Redmond, Washington).

## Results

During the study period, 253 patients presented with proximal femur fractures and met the inclusion criteria. The average patient age was 79.72 ± 10.54 and 64.8% were females. The mean Charlson Comorbidity Index (CCI) was 5.05 ± 2.00. The mean hospitalization period in days was 7.48 ± 5.50, and the time to operation was 28.18 ± 24.52. A significant difference between orthopedic and geriatric patients was observed in the age and CCI hospitalization length. Demographic data regarding the entire cohort and a comparison of the cohorts of the geriatric and orthopedic departments are presented in Table 1.

**Table 1:** Demographic data

Demographics		Total	Geriatric department	Orthopedic department	Sig.
Age		79.75 (10.54)	82.80 (9.08)	78.72 (10.82)	0.007
Sex (Female)		164	46 (71.9%)	118 (62.4%)	0.17
CCI		5.05 (2.00)	5.61 (1.94)	4.86 (1.99)	0.009
Place of residence	Alone	72 (28.5%)	26 (40.6%)	46 (24.3%)	
	Home with family	168 (66.4%)	34 (53.1%)	134 (70.9%)	
	Nursing home	13 (5.1%)	4 (6.6%)	9 (4.8%)	
Marital status	Single	6 (2.4%)	0 (0%)	6 (3.2%)	
	Married	119 (47.0%)	25 (39.1%)	94 (49.7%)	
	Divorced	36 (14.2%)	10 (15.6%)	26 (13.8%)	
	Widower	92 (36.4%)	29 (45.3%)	63 (33.3)	
Hospitalization length in days		7.48 (5.50)	10.22 (6.739)	6.55 (4.68)	0.001
Time to operation in hours		29.18 (24.51)	27.33 (19.55)	29.81 (26.00)	0.49

Twenty-three patients passed the 48 hours of operation limit. The main reasons for the delay were sepsis, pneumonia, patients on novel oral anticoagulants (NOACs), cardiovascular symptoms (acute coronary syndrome, myocardial infarction), cerebral vascular accident, and patients' choice.

There was no significance in the fracture and operation type. There was a tendency to do more hemiarthroplasty operations rather than total hip replacements (THR) in the geriatric department, while the opposite was found in the orthopedic department (Table 2).

**Table 2:** Comparison of the fracture and operation types between geriatric and orthopedic departments DHS - dynamic hip screw; IMN - intramedullary nailing; THR - total hip replacements

		Total	Geriatric department	Orthopedic department	Sig.
Fracture type	Transcervical	93 (36.7%)	22 (34.4%)	71 (37.5%)	0.89
	Base of neck	18 (7.1%)	5 (7.8%)	13 (6.9%)	
	Trochanteric	142 (56.1%)	37 (57.8%)	105 (55.6%)	
Operation type	DHS	19 (7.5%)	5 (7.8%)	14 (7.4%)	0.64
	IMN	136 (53.6%)	35 (54.7%)	101 (53.4%)	
	Hemiarthroplasty	42 (16.6%)	13 (20.3%%)	29 (15.3%)	
	THR	56 (22.1%)	11 (17.2%)	45 (23.8%)	

The mean mortality was 147.55 ± 128.31 days during one-year follow-up; significance was observed comparing geriatrics versus orthopedics (91.53 ± 112.24 and 176.52 ± 128.193; p=0.04). The mean mortality rate after surgery in the first year of follow-up was 16.5%, with increased mortality in the geriatric department. During hospitalization, mortality rates in geriatrics 6.3% versus 0.5% in orthopedics (p=0.03; Table 3).

**Table 3:** Mortality geriatrics versus orthopedic departments during the follow-up period

Department	During hospitalization	Up to 30 days	Up to 90	1 year
Geriatrics	4 (6.3%)	6 (9.5%)	10 (15.9%)	15 (23.8%)
Orthopedics	1 (0.5%)	5 (2.6%)	9 (4.8%)	26 (13.9%)
Total	5 (2%)	11 (4.4%)	19 (7.6%)	41 (16.5%)

At the end of hospitalization from both departments, 78.2% of patients had been released to rehabilitation centers. Of the geriatric patients, 82.5% were sent to the orthopedic department and 65.1% were sent to rehabilitation centers. The opposite was seen for sending patients to home rehabilitation 7.4% of orthopedic patients versus 17.5% of geriatric patients (Table 4).

**Table 4:** Rehabilitation placement after discharge

	Total	Geriatrics	Orthopedics
Rehabilitation	197 (78.2%)	41 (65.1%)	156 (82.5%)
Home rehabilitation	25 (9.9%)	11 (17.5%)	14 (7.4%)
Other in hospital departments	30 (11.9%)	11 (17.5%)	19 (10.1%)

Among the excluded, 26 patients had conservative nonsurgical treatment. The analysis for these patients was made regardless of the hospitalization placement. The mean age was 82.73 ± 10.23. 69% were females. The mean CCI was 6.04 ± 2.27. The hospitalization time was 7.58 ± 7.97 days. Among the patients, 15.4 % had been discharged from the emergency department; orthopedic and geriatric departments received an equal amount of 38.5 % of the patients. The mortality average of patients without operation was 50.07 ± 58.75 days; 57.7% died during the first year of follow-up, five patients (19.2%) died during the hospitalization, and 15.4% during the first 30 days after hospitalization.

## Discussion

Our study showed that patients hospitalized in the geriatric department were older patients with more comorbidities and higher CCI in the geriatric department rather than in the orthopedic ward. Time to surgery was shorter in the geriatric department patients, showing the potency of geriatric physicians in managing preoperative-related problems. Ortho-geriatric collaboration has previously been shown to reduce the time to operation [17,18]. In our study, the time to surgery was similar in both departments, slightly faster in the geriatric department. It was much better in both departments than the suggested 48-hour limit [19,20]. A similar percentage of fractures and operation types were performed in both departments. The average hospitalization in the geriatric department was around 3.6 days longer than in the orthopedic department. Although patients in the geriatric department stayed longer, a significantly greater percentage of them were released to their homes for rehabilitation than in the orthopedic department. Our results suggest that meticulous post-operative hip fracture management takes a few more days of in-hospital care but enables more patients to be more independent back home. Even though most patients in both departments were sent to rehabilitation centers, the ability to send elderly hip fracture patients back home may impact the overall well-being and cost of management of these patients [1]. 

The mortality rates during hospitalization showed increased mortality in the geriatric department; this may be explained as the patients in the geriatric department were initially (before the fracture) sicker than those in the orthopedic department. The overall one-year mortality showed similar results, with higher mortality rates in the geriatric department. Patients who underwent surgery had an average of 16.5% one-year mortality versus 57.7% in the non-operated patients. This may be explained by the fact that some patients were too sick to be operated on and that surgery is more beneficial than a nonoperative treatment approach. This corresponds to similar data in previous studies [21,22]. 

This study has several limitations. It is a nonrandomized, retrospective study, which may have influenced the final results of our research. It is a single-center setting, and there was not an equal number of patients in each group. As shown, the geriatric ward patients were older, which could have introduced selection bias. The proportion of male patients was under-represented, and we could not receive information about the patients' causes of death. We could not exclude the possibility that confounding factors like lack of information on preoperative functional level or socioeconomic factors influenced our findings. Despite these limitations, to the best of our knowledge, our study is the first to directly compare two orthogeriatric care models for proximal hip fractures in the same hospital [23]. 

Elderly patients with hip fractures require efficient multidisciplinary care. The standard and historical approach of hospitalizing patients in the orthopedic department, with geriatric consultations based on demand, needs to be re-evaluated. However, improving standards of care in a hospital framework is a complex process that requires urgent attention [24]. The involvement of the national health system ministry is crucial for the proper implementation and adjustment of new regulations to develop and implement these changes [25]. The capacity of hospitals for organizational change should be evaluated to ensure the successful implementation of these improvements [26].

We believe that the standard of care for patients with proximal hip fractures should involve hospitalization in the geriatric department. This approach, which includes daily follow-ups by orthopedic physicians before and after surgery, has the potential to significantly improve patient outcomes. Our study aimed to compare these patients between the two departments to improve the quality standards of care and make the necessary changes to the healthcare system. We emphasize the importance of a collaborative approach, which involves orthopedic surgeons and geriatric specialists working together to provide the best possible care for these patients. Geriatric medicine, with its focus on team building, functional assessment, and aging physiology, plays a crucial role in this collaborative approach [14].

## Conclusions

The geriatric department manages elderly patients with hip fractures well. However, the department's population has higher CCI, comorbidities, and mortality rates. They also showed significantly longer hospitalization periods and a higher chance of discharge home than the orthopedic department.

## References

[1] Barnea R, Weiss Y, Abadi-Korek I, Shemer J (2018). The epidemiology and economic burden of hip fractures in Israel. Isr J Health Policy Res.

[2] Johnell O, Kanis JA (2006). An estimate of the worldwide prevalence and disability associated with osteoporotic fractures. Osteoporos Int.

[3] Nikitovic M, Wodchis WP, Krahn MD, Cadarette SM (2013). Direct health-care costs attributed to hip fractures among seniors: a matched cohort study. Osteoporos Int.

[4] Ahlborg HG, Rosengren BE, Järvinen TL, Rogmark C, Nilsson JA, Sernbo I, Karlsson MK (2010). Prevalence of osteoporosis and incidence of hip fracture in women - secular trends over 30 years. BMC Musculoskelet Disord.

[5] Tanner DA, Kloseck M, Crilly RG, Chesworth B, Gilliland J (2010). Hip fracture types in men and women change differently with age. BMC Geriatr.

[6] Sullivan KJ, Husak LE, Altebarmakian M, Brox WT (2016). Demographic factors in hip fracture incidence and mortality rates in California, 2000-2011. J Orthop Surg Res.

[7] Sterling RS (2011). Gender and race/ethnicity differences in hip fracture incidence, morbidity, mortality, and function. Clin Orthop Relat Res.

[8] Kanis JA, Oden A, Johnell O, De Laet C, Jonsson B, Oglesby AK (2003). The components of excess mortality after hip fracture. Bone.

[9] Roche JJ, Wenn RT, Sahota O, Moran CG (2005). Effect of comorbidities and postoperative complications on mortality after hip fracture in elderly people: prospective observational cohort study. BMJ.

[10] Morri M, Ambrosi E, Chiari P, Orlandi Magli A, Gazineo D, D' Alessandro F, Forni C (2019). One-year mortality after hip fracture surgery and prognostic factors: a prospective cohort study. Sci Rep.

[11] Loggers SA, Van Lieshout EM, Joosse P, Verhofstad MH, Willems HC (2020). Prognosis of nonoperative treatment in elderly patients with a hip fracture: a systematic review and meta-analysis. Injury.

[12] Donohoe E, Roberts HJ, Miclau T, Kreder H (2020). Management of lower extremity fractures in the elderly: a focus on post-operative rehabilitation. Injury.

[13] Dyer SM, Crotty M, Fairhall N, Magaziner J, Beaupre LA, Cameron ID, Sherrington C (2016). A critical review of the long-term disability outcomes following hip fracture. BMC Geriatr.

[14] Grigoryan KV, Javedan H, Rudolph JL (2014). Orthogeriatric care models and outcomes in hip fracture patients: a systematic review and meta-analysis. J Orthop Trauma.

[15] Baroni M, Serra R, Boccardi V (2019). The orthogeriatric comanagement improves clinical outcomes of hip fracture in older adults. Osteoporos Int.

[16] Kammerlander C, Roth T, Friedman SM (2010). Ortho-geriatric service - a literature review comparing different models. Osteoporos Int.

[17] Biber R, Singler K, Curschmann-Horter M, Wicklein S, Sieber C, Bail HJ (2013). Implementation of a co-managed geriatric fracture center reduces hospital stay and time-to-operation in elderly femoral neck fracture patients. Arch Orthop Trauma Surg.

[18] González-Montalvo JI, Alarcón T, Mauleón JL, Gil-Garay E, Gotor P, Martín-Vega A (2010). The orthogeriatric unit for acute patients: a new model of care that improves efficiency in the management of patients with hip fracture. Hip Int.

[19] Brox WT, Roberts KC, Taksali S (2015). The American Academy of Orthopaedic Surgeons evidence-based guideline on management of hip fractures in the elderly. J Bone Joint Surg Am.

[20] Lewis PM, Waddell JP (2016). When is the ideal time to operate on a patient with a fracture of the hip?: a review of the available literature. Bone Joint J.

[21] Prommik P, Tootsi K, Saluse T, Märtson A, Kolk H (2021). Nonoperative hip fracture management practices and patient survival compared to surgical care: an analysis of Estonian population-wide data. Arch Osteoporos.

[22] van de Ree CL, De Jongh MA, Peeters CM, de Munter L, Roukema JA, Gosens T (2017). Hip fractures in elderly people: surgery or no surgery? A systematic review and meta-analysis. Geriatr Orthop Surg Rehabil.

[23] Hu F, Jiang C, Shen J, Tang P, Wang Y (2012). Preoperative predictors for mortality following hip fracture surgery: a systematic review and meta-analysis. Injury.

[24] Sabharwal S, Wilson H (2015). Orthogeriatrics in the management of frail older patients with a fragility fracture. Osteoporos Int.

[25] Moldovan F, Moldovan L (2023). Fair healthcare practices in orthopedics assessed with a new framework. Healthcare (Basel).

[26] Janssens S, Deschodt M, Dejaeger M (2023). From research to daily clinical practice: implementation of orthogeriatric co-management in the trauma ward. Front Health Serv.

